# A prospective study of predictors of return to work after surgery for ulnar nerve entrapment

**DOI:** 10.1038/s41598-025-21589-z

**Published:** 2025-09-29

**Authors:** Alice Giöstad, Kamil Piotr, Malin Zimmerman, Erika Nyman

**Affiliations:** 1 Division of Family Medicine and Primary Care, Department of Neurobiology, Care Sciences and Society, Karolinska Institute, Stockholm, Sweden; 2 Academic Primary Health Care Centre, Region Stockholm, Stockholm, Sweden; 3 Department of Biomedical and Clinical Sciences, Linköping University, Linköping, Sweden; 4 Department of Translational Medicine – Hand Surgery, Lund University, Skåne University Hospital, 205 02 Malmö, Sweden; 5 Department of Orthopaedics, Helsingborg Hospital, Helsingborg, Sweden; 6 Department of Hand Surgery, Plastic Surgery and Burns, Linköping University Hospital, Linköping, Sweden

**Keywords:** Diseases, Health care, Medical research, Risk factors

## Abstract

Ulnar nerve entrapment at the elbow (UNE) is the second most common compression neuropathy in the upper limb. Immediate return-to-work (RTW) after surgery is seldom possible due to restrictions and recovery demands. We aimed to explore RTW after UNE surgery and investigate factors contributing to variations. A prospective study with patients undergoing primary UNE surgery was conducted. Treatment decisions were based on a novel five-grade clinical scale. In total, 65 patients (25 women and 40 men) with a mean age of 51 ± 16 years were included. Simple decompression was the predominant procedure (n = 57, 88%). Manual laborers (n = 28, 43%) had the longest sick leave (7 weeks, IQR [3]) within the working population (n = 49) compared to non-manual laborers (4 weeks, IQR [6]; p = 0.003). Manual labor, smoking, and preoperative pain (evaluated with the Swedish version of the Patient-Rated Ulnar Nerve Evaluation, PRUNE-S) predicted prolonged sick leave. The type of surgery did not predict RTW variations in the multivariate linear model itself but modified the impact of other predictors. In conclusion, patients with surgically treated UNE have varying sick leave needs, influenced by individual and work-related factors. Incorporating biopsychosocial aspects should be a focus of further research in UNE management.

## Introduction

The estimated incidence rate of ulnar nerve entrapment at the elbow (UNE) varies between 20–36 cases per 100,000 person-years^1,2^. It can occur at any age, although it is most prevalent in patients of working age^3^. The etiology of UNE is often idiopathic, with conflicting evidence on risk factors. Smoking, heavy manual work, and a low level of education are suggested to increase the risk of developing UNE, while the influence of sex varies across studies^3–5^. Patients with UNE may also present with other concomitant nerve compressions in the same or opposite arm^6^.

Immediate return to work (RTW) after UNE surgery may not be possible due to functional restrictions of the affected arm and the time needed for healing and recovery. The Swedish National Board of Health and Welfare (*Socialstyrelsen*) provides guidelines (*Försäkringsmedicinskt beslutsstöd*)^7^ for physicians to assess the length of sick leave for specific medical conditions. Recommendations after surgery for UNE have recently been published in Sweden, highlighting the potential need for up to 8 weeks of sick leave, depending on the type of surgery and the patient’s work tasks^8^. For comparison, 4–8 weeks are recommended for carpal tunnel release in the same guidelines^9^. Although sick leave after surgery may be necessary, prolonged sick leave can negatively influence the surgical outcome^10,11^.

While the guidelines provide useful insights into sick leave management for UNE, more detailed studies are needed. Research on RTW after surgery for idiopathic UNE is scarce. A recent systematic review and meta-analysis revealed no difference in RTW based on surgical techniques; however, none of the studies assessing RTW as an outcome included anterior subcutaneous transposition as a surgical technique^12^. In a previously published retrospective study including patients having surgery for UNE during 2004–2014, we reported a median RTW time of 6 weeks after simple decompression, regardless of occupation, and 8 weeks after transposition or revision surgery, which is in accordance with the new guidelines. Prolonged RTW (defined as > 6 weeks) was associated with younger age, manual labor, and unemployment^13^. Prospective studies that include both pre- and postoperative data, as well as information on patient characteristics, are needed to increase understanding of outcomes of UNE surgery. This study aims to explore RTW after surgery for UNE in a prospective study design and investigate predictors for sick leave variations. We hypothesized that age, tobacco usage, manual labor, type of surgery, preoperative sick leave, previous or current pain issues, and preoperative symptoms, including pain, could influence RTW after surgery.

## Methods

**Fig. 1:**
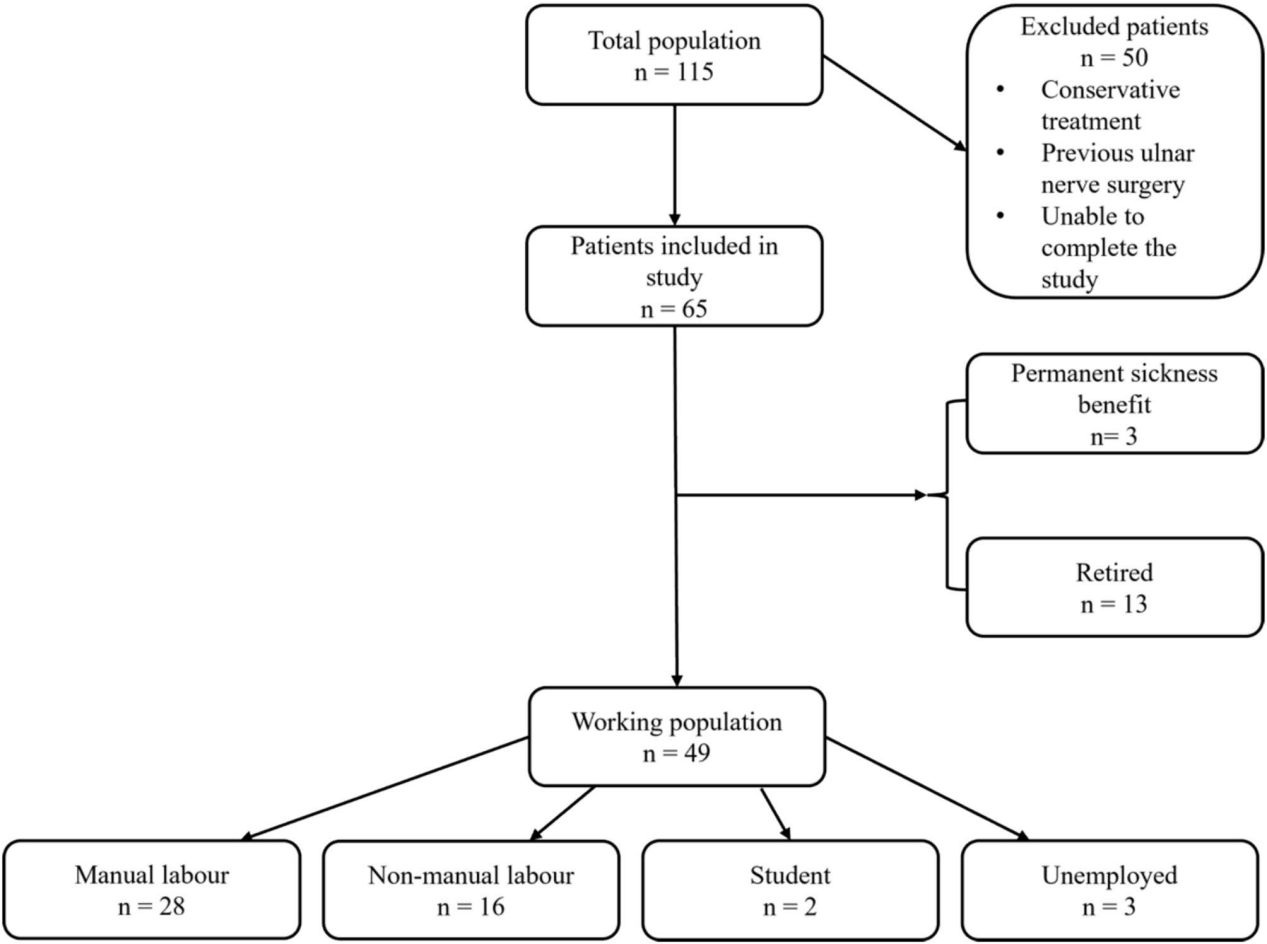
Flowchart of study population and overview of included patients.

A prospective cohort study was conducted between 2020 and 2024 at the Department of Hand Surgery, Plastic Surgery and Burns at Linköping University Hospital, Östergötland, Sweden. Patients included were ≥ 18 years old, referred to the department due to UNE, and followed for 12 months postoperatively. The cohort consisted of 65 surgically treated patients, as illustrated in the flowchart (Fig. 1). Patients who did not understand spoken and/or written Swedish, had previously undergone ulnar nerve surgery, only received conservative treatment, or were judged unable to complete preoperative examinations, surgery, or postoperative rehabilitation, were excluded from this study.

Occupations were categorized as manual or non-manual labor based on the Swedish Standard Classification of Occupations (SSYK 2012), which is based on the International Standard Classification of Occupations (ISCO-08)^14^. The working population consisted of employed patients (manual or non-manual labor), students, and those who were unemployed (Fig. 1). Some patients were on preoperative sick leave but otherwise employed and able to return to work postoperatively, thereby contributing to the sick leave data. Demographic and comprehensive medical information were collected (i.e., age, sex, history of previous ulnar nerve surgeries, tobacco use, dominant hand, symptom side, duration of symptoms, comorbidities, and preoperative sick leave).

Preoperative clinical assessments served as a basis for a novel five-grade symptom grading system (Table 1) developed by Nyman et al. (to be published). An algorithm guided successive treatment interventions: patients with grade 1 received a follow-up with an occupational therapist at 3 months; those with grades 2–3 were planned for a follow-up with a physician at 3 months; and patients with grades 4–5 were offered surgery, with continuous follow-ups at 6 weeks, 3, and 12 months. Patients classified as grade 2–3 with no change at 3-month follow-up were randomized to continue conservative treatment or undergo surgery, while those who worsened were directly offered surgery. Similarly, grade 4 patients were offered surgery if their condition was unchanged or worsened at follow-up. Simple decompression was the primary procedure. Anterior subcutaneous transposition was performed in patients with pre- or perioperative subluxation of the ulnar nerve. Patients classified as grade 5 were randomized to either simple nerve decompression or decompression with an additional nerve transfer (end-to-side) of the anterior interosseus nerve to the motor branch of the ulnar nerve.

**Table 1:** The grading system used to assess the severity of symptoms.

Grading system	Definition
Grade 1	Intermittent sensory symptoms, no motor symptoms
Grade 2a	Intermittent sensory symptoms with subjective motor symptoms, no objective motor impairment
Grade 2b	Constant sensory symptoms, no motor symptoms
Grade 3	Constant sensory symptoms with subjective motor symptoms, no objective motor impairment
Grade 4	Constant sensory symptoms with objective motor impairment but no atrophy
Grade 5	Constant sensory symptoms with motor impairment and atrophy

The follow-up assessments (6 weeks, 3, and 12 months postoperatively) included both patient-rated outcome measures (PROMs) as well as specific items for this study. At each follow-up, patients were asked to evaluate the surgical outcome on a 4-level scale: *completely recovered, improved, unchanged, or worsened.* Two questions regarding RTW were asked, namely “Have you returned to work yet?” and “Have you been on sick leave?”, followed by the duration in whole weeks. The primary outcome measure was full-time sick leave. In cases where sick leave duration was reported in months, the number of weeks was calculated by assumption: 1 month = 30 days divided by 7, and rounded to the nearest whole number. In cases with sick leave < 7 days, the following assumptions were made: 0–3 days = 0 weeks; 4–6 days = 1 week. If the duration exceeded one year, it was calculated as 12 months, due to the follow-up limit of the study. The Patient-Rated Ulnar Nerve Evaluation (PRUNE) is a specific PROM for assessing pain, symptoms, and functional disability in patients with UNE^15^. The Swedish version comprises six questions about pain, two about sensory symptoms, and twelve related to different activities (Appendix 1)^16^. Patients score their difficulties on a scale from 0–10 for each question. The total score is calculated and ranges from 0–100, where higher scores indicate greater severity or functional disability. Changes in score reflect changes in symptoms^17^. Patients in this study were asked to fill in the PRUNE-S during the follow-up assessments.

This study was conducted in line with the ethical principles in the 7^th^ revision (2013) of the Declaration of Helsinki, with ethical approval granted by the National Ethics Review Board (registration number 2019–05-158). Patients received detailed information about the study, both verbally and in writing, and provided informed consent in both formats. Surgical treatments adhered to evidence-based practices (EBP) and were performed in line with proven clinical experience. Participation was voluntary with the right to withdraw at any time. Those who chose not to participate would still receive conventional care based on standard practices of the hospital.

Normality was assessed using the Shapiro–Wilk test. Normally distributed values are presented as mean ± SD; non-normally distributed values are presented as median [IQR]. T-tests were used for the age variable. The Chi-Square or Fisher’s Exact test and the Mann–Whitney U test were used to compare groups and analyze dichotomized variables, respectively. The Kruskal–Wallis test and the Mann–Whitney U test were subsequently used for non-parametric data. The PRUNE-score was dichotomized into high versus low scores based on the cohort medians (since no standardized cut-off exists). The same dichotomization was done for the age variable.

To investigate the impact of various variables contributing to differences in sick leave patterns, a multivariate, manually reduced, linear regression analysis was conducted. The analysis began with a full model including eleven independent variables. Variables were chosen manually, and those with the highest p-value were removed from the regression, one at a time. Changes in the significance of the remaining variables were observed, along with their individual effect or interactions, by looking at changes in unstandardized B coefficients. This stepwise process produced a final model with the highest explanatory power. The significance was set at p < 0.05. A post hoc power analysis was conducted in G*Power 3.1.9.7 for a linear multiple regression (fixed model, single predictor). With an alpha level of 0.05, 49 participants, 6 predictors, and an observed effect size of *f*^*2*^ = 4.9, the analysis yielded a power of 1.00.

All data management and statistical analyses were performed using the software SPSS (IBM corp. Released 2023. IBM SPSS Statistics for Windows, Version 29.0.0.0 Armonk, NY: IBM Corp).

## Results

### Basic demographics and sex differences

In total, 65 patients (25 women and 40 men; mean age at surgery 51 ± 16 years) in the population underwent surgery (Fig. 1). Simple decompression was the predominant procedure (57/65; 88%), compared to transposition surgery (8/65; 12%). The working population consisted of 49 patients, classified as either manual or non-manual laborers, students, or unemployed individuals (Table 2). Preoperative sick leave was reported by six patients (6/61; 10%), of whom five were men. Manual occupations were the most common occupations among both sexes. When comparing men and women, no statistically significant differences were found for age, type of occupation, type of surgery, preoperative sick leave, or preoperative McGowan grade. The majority of patients were non-smokers (57/65; 88%), with smoking being more common among women (p = 0.047, Fisher’s Exact test) (Table 2). Snus use was observed in 21/64, (33%) patients and was more common among men (p = 0.006, Chi Square). There was a significant sex difference regarding the preoperative symptom grading system, where more men than expected were classified as grade 5 (p = 0.016, Chi Square).

**Table 2:** Basic characteristics for the whole population of patients treated surgically for ulnar nerve entrapment at the elbow and sick leave length for the working population.

		Total population n = 65	Sick leave, weeks median [IQR] n = 49	p-value
Age	Higher age group (range 51–85 years)	32 (49)	7 [6]	0.802*
	Lower age group (range 24–50 years)	33 (51)	6 [4]	
Sex	Women	25 (38)	6 [5]	0.887*
	Men	40 (62)	6 [4]	
Preoperative symptom grade 1–5	Grade 1	2 (4)	6 [0]	0.830**
	Grade 2a	9 (14)	7 [5]	
	Grade 2b	5 (8)	4 [6]	
	Grade 3	9 (14)	8 [5]	
	Grade 4	18 (28)	6 [5]	
	Grade 5	22 (34)	7 [7]	
Preoperative McGowan	Grade 1	16 (25)	6 [4]	0.788**
	Grade 2	27 (42)	6 [5]	
	Grade 3	21 (33)	7 [7]	
Smoking	Yes	8 (12)	6 [19]	0.688*
	No	57 (88)	6 [4]	
Snus user ^a)^	Yes	21 (33)	6 [5]	0.105*
	No	43 (67)	7 [5]	
Previous or current pain issues	Yes	15 (23)	5 [5]	0.265*
	No	48 (74)	7 [3]	
Previous contact with a pain clinic ^b)^	Yes	5 (8)	6 [−]	0.913*
	No	58 (92)	6 [4]	
Occupation	Non-manual labor	16 (25)	4 [5]	0.025** ^(All groups)^ 0.003* ^non-manual vs manual (post-hoc)^
	Manual labor	28 (43)	7 [3]	
	Student ^d)^	2 (3)	4 [–]	
	Unemployed ^e)^	3(5)	3 [−]	
	Permanent sickness benefit	3 (5)	N/A	
	Retired	13 (20)	N/A	
Preoperative sick leave ^c)^	Yes	6 (10)	8 [11]	0.200*
	No	55 (90)	6 [5]	
Type of surgery	Simple decompression	57 (88)	6 [4]	0.851*
	Transposition	8 (12)	6 [2]	
Patient reported outcome 6w postoperatively	Improved (completely recovered or improved)	58 (89)	6 [4]	0,590*
	Not improved (unchanged or worse)	7 (11)	6 [9]	

* Mann–Whitney U, ** Kruskal–Wallis. Values in parentheses represent valid percentages. Percentages have been rounded and may not sum to 100%. ^a)^ n = 64 ^b)^ n = 63 ^c)^ n = 61 ^d)^ Since there were only 2 students, the IQR could not be calculated ^e)^. Since there were only 3 unemployed patients, the IQR could not be calculated. Statistically significant values (p < 0.05) are presented in **bold**.

### Postoperative sick leave

**Fig. 2:**
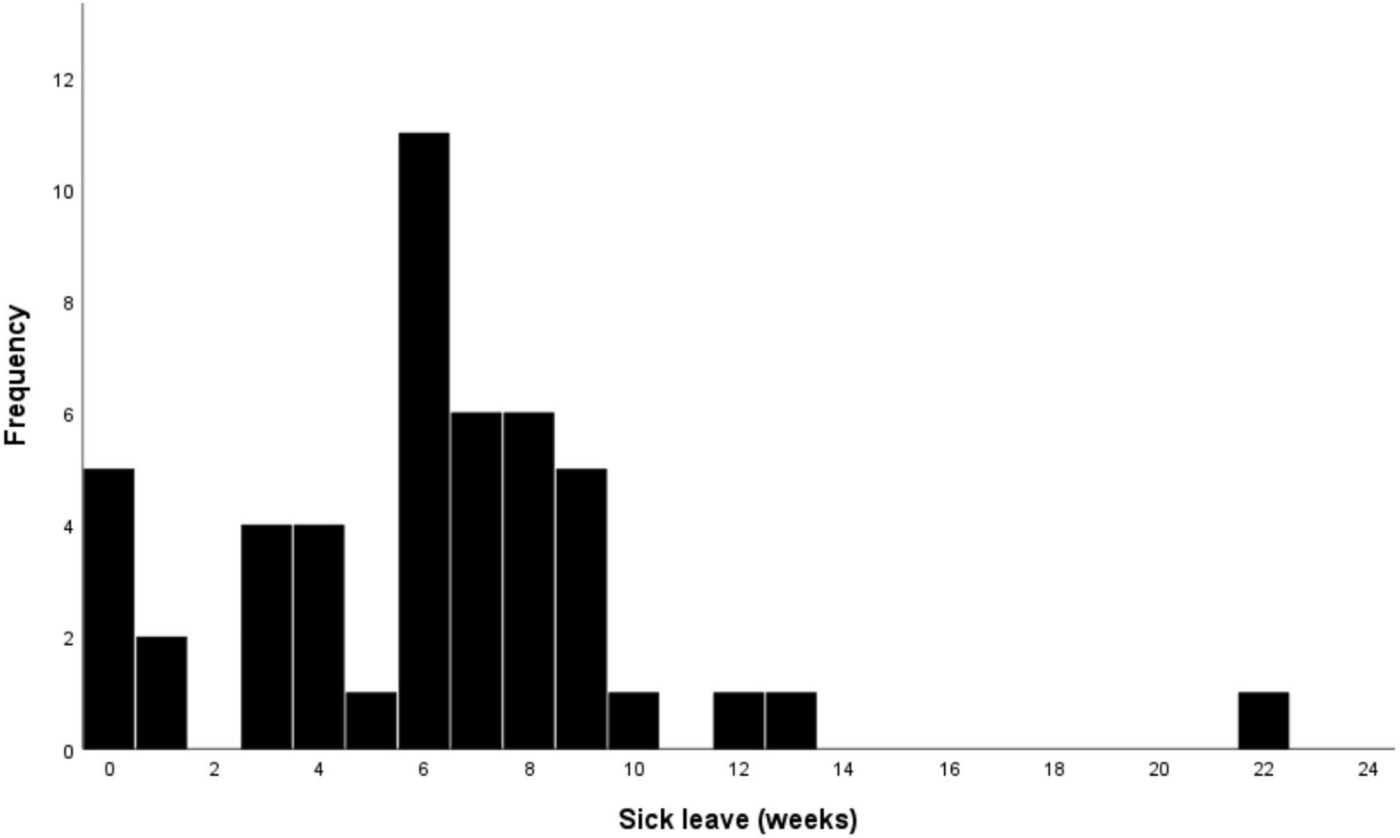
Distribution of sick leave in weeks across the working population in patients treated surgically for ulnar nerve entrapment at the elbow. Outlier (sick leave = 52 weeks) not shown, but included in the statistical analyses.

The median duration of full-time sick leave was 6 weeks [IQR 4] for the entire population eligible for work (manual laborers, non-manual laborers, students, and unemployed individuals). The distribution of sick leave in weeks can be seen in Fig. 2.

**Fig. 3:**
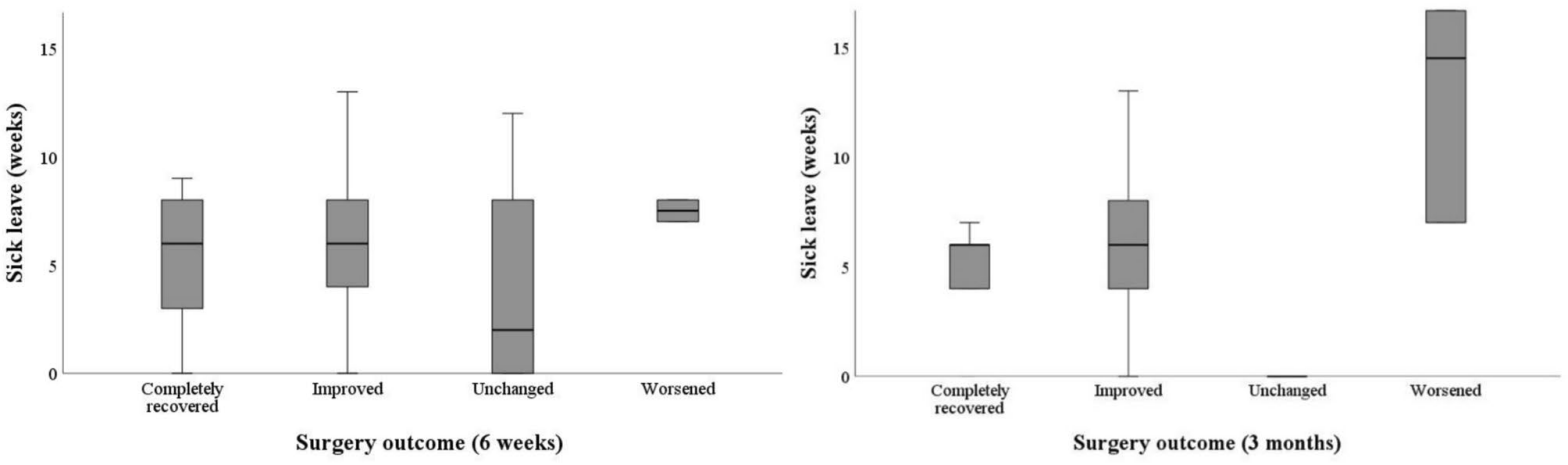
Sick leave length (in weeks) stratified by patients’ perceived surgical outcomes at two postoperative timepoints: 6 weeks (left) and 3 months (right). The margins of each box represent the interquartile range [IQR], with the median sick leave marked by a horizontal line within each box. Sick leave data represent the total number of weeks for each patient over the 12-month study period. Patient may thus still be on sick leave at the time of follow-ups (e.g., more than 6 weeks at 6-weeks etc.)

We found no differences in length of sick leave when comparing age groups, sex, preoperative symptom grade, McGowan grade, smoking status, snus use, previous or current pain issues, comorbidities (data not shown), preoperative sick leave, type of surgery, or patient reported surgical outcome (Table 2, Fig. 3).

#### PRUNE-S

Preoperative PRUNE-S responses were obtained from 62 patients, with a median score of 51 [IQR 23] (Table 3). At both the 3-month and 12-month postoperative follow-ups, the median scores showed a decreasing trend for both the total score (PRUNE-S sum) as well as for the subscales (Table 3).

**Table 3:** PRUNE-S scores with subscales across preoperative and postoperative timelines in patients treated surgically for ulnar nerve entrapment at the elbow.

PRUNE-S timeline	Total score
**Preoperative PRUNE-S sum (n = 62)**	51 [23]
Pain subscale	34 [19]
Sensory symptom subscale	15 [7]
Specific activities subscale	44 [21]
Usual activities subscale	14 [15]
**3-months postoperative PRUNE-S sum (n = 46)**	21 [33]
3-months pain subscale	12 [21]
3-months sensory symptom subscale	5 [9]
3-months specific activities subscale	18 [31]
3-months usual activities subscale	4 [13]
**12-months postoperative PRUNE-S sum (n = 34)**	16 [28]
12-months pain subscale	6 [24]
12-months sensory symptom subscale	5 [9]
12-months specific activities subscale	12 [31]
12-months usual activities subscale	3 [10]

PRUNE-S = Patient-Rated Ulnar Nerve Evaluation Swedish version. Total score presented as median [IQR].

When analyzing the dichotomized preoperative PRUNE-S sum, no significant difference in sick leave duration was observed between patients with higher scores (above the median) and those with lower scores (below the median, data not shown).

The working population was stratified based on whether their pain subscale scores were above or below the median. Patients with higher pain scores had a median sick leave of seven weeks, while those with lower scores had a median of six weeks, this difference was however, non-significant (p = 0.056, Mann–Whitney U test).

### History of sick leave and comorbidity

A few patients (6/61; 10%) reported being on sick leave preoperatively due to all causes. This group had a longer postoperative sick leave length compared to those without a preoperative history of sick leave, however, this difference was not statistically significant (median 8 weeks [IQR 11] vs 6 weeks [IQR 5]; p = 0.20, Mann–Whitney U test, Table 2). A large proportion of patients (18/65; 28%) had previously undergone surgery for ipsilateral carpal tunnel syndrome (CTS), and the same percentage (18/65; 28%) had undergone the procedure for contralateral CTS. At the time of inclusion in the study19/65 (30%) of patients reported ongoing problems with ipsilateral CTS, and 17/65 (27%) reported issues with their contralateral CTS. Patients with previous surgery for CTS may not be the same as those who reported ongoing CTS symptoms at inclusion time. Other commonly reported comorbidities included shoulder (31/65; 48%) and neck problems (28/65; 44%). These problems were more prevalent in men than in women (not statistically significant). Additionally, 22/65 (35%) of the patients had a history of cardiovascular disease or hypertension. A substantial portion (15/65; 24%) of the patients had reported some psychiatric condition or addiction, and the same percentage had present or recurrent pain issues. In all these categories, a higher prevalence of comorbidities was observed in men compared to women, data not shown.

### Regression analyses RTW

The linear regression model revealed that smoking, manual labor, and preoperative pain (measured with PRUNE-S) were the only significant predictors of sick leave patterns across all models (Table 4). Age, sex, preoperative sick leave, preoperative symptom grade, snus use, previous or current pain issues, and preoperative PRUNE-S sum were not significant predictors of sick leave length (Table 4). Additional models resulted in a decrease in adjusted R^2^ (data not shown). Despite including non-significant variables, Model 6 presented the highest explanatory power and was chosen as the final model.

**Table 4:** Linear regression model analyzing the effect of selected variables on postoperative sick leave (weeks) in patients treated surgically for ulnar nerve entrapment at the elbow.

Model	Variable	Unstandardized B	95% CI	p-value	Adj. R^2^
Model 1	Age	-0.055	[-0.273, 0.163]	0.610	0.316
	Sex (female is reference)	2.063	[-2.819, 6.945]	0.395	
	Smoking status (smoking is reference)	-12.546	[-19.551, -5.541]	** < 0.001**	
	Snus use (current use is reference)	4.498	[-0.256, 9.252]	0.063	
	Preoperative sick leave (yes is reference)	2.636	[-4.967, 10.238]	0.485	
	Previous or current pain issues (yes is reference)	-0.009	[-5.743, 5.726]	0.998	
	Preoperative symptom grade 1–5 (grade 1 is reference)	-0.687	[-2.442, 1.067]	0.430	
	Labor (non-manual labor is reference)	6.154	[0.969, 11.339]	**0.022**	
	Type of surgery (SD is reference)	-5.314	[-12.283, 1.656]	0.130	
	Dichotomized PRUNE-S sum	-2.087	[-8.114, 3.940]	0.485	
	Dichotomized PRUNE-S pain	7.243	[1.306, 13.180]	**0.018**	
Model 2	Age	-0.055	[-0.267, 0.157]	0.600	0.337
	Sex (female is reference)	2.063	[-2.709, 6.834]	0.385	
	Smoking status (smoking is reference)	-12.545	[-19.411, -5.679]	** < 0.001**	
	Snus use (current use is reference)	4.497	[-0.088, 9.081]	0.054	
	Preoperative sick leave (yes is reference)	2.634	[-4.774, 10.042]	0.474	
	Preoperative symptom grade 1–5 (grade 1 is reference)	-0.688	[-2.364, 0.988]	0.409	
	Labor (non-manual labor is reference)	6.152	[1.218, 11.087]	**0.016**	
	Type of surgery (SD is reference)	-5.314	[-12.165, 1.538]	0.124	
	Dichotomized PRUNE-S sum	-2.086	[-7.965, 3.793]	0.475	
	Dichotomized PRUNE-S pain	7.244	[1.414, 13.073]	**0.016**	
Model 3	Sex (female is reference)	1.908	[-2.769, 6.586]	0.412	0.352
	Smoking status (smoking is reference)	-12.457	[-19.232, -5.683]	** < 0.001**	
	Snus use (current use is reference)	4.501	[-0.028, 9.030]	0.051	
	Preoperative sick leave (yes is reference)	2.826	[-4.456, 10.107]	0.435	
	Preoperative symptom grade 1–5 (grade 1 is reference)	-0.902	[-2.346, 0.542]	0.213	
	Labor (non-manual labor is reference)	6.246	[1.385, 11.108]	**0.013**	
	Type of surgery (SD is reference)	-5.594	[-12.278, 1.090]	0.098	
	Dichotomized PRUNE-S sum	-2.060	[-7.866, 3.747]	0.476	
	Dichotomized PRUNE-S pain	7.410	[1.686, 13.135]	**0.013**	
Model 4	Sex (female is reference)	2.140	[-2.454, 6.734]	0.351	0.361
	Smoking status (smoking is reference)	-12.109	[-18.757, -5.461]	** < 0.001**	
	Snus use (current use is reference)	4.446	[-0.043, 8.936]	0.052	
	Preoperative sick leave (yes is reference)	2.438	[-4.702, 9.579]	0.492	
	Preoperative symptom grade 1–5 (grade 1 is reference)	-1.022	[-2.414, 0.371]	0.145	
	Labor (non-manual labor is reference)	6.256	[1.434, 11.078]	**0.013**	
	Type of surgery (SD is reference)	-5.614	[-12.243, 1.015]	0.094	
	Dichotomized PRUNE-S pain	6.044	[1.843, 10.246]	**0.006**	
Model 5	Sex (female is reference)	1.536	[-2.7.44, 5.815]	0.472	0.359
	Smoking status (smoking is reference)	-11.715	[-18.082, -5.349]	** < 0.001**	
	Snus use (current use is reference)	4.066	[-0.262, 8.395]	0.065	
	Preoperative symptom grade 1–5 (grade 1 is reference)	-1.148	[-2.463, 0.167]	0.085	
	Labor (non-manual labor is reference)	5.259	[1.113, 9.405]	**0.014**	
	Type of surgery (SD is reference)	-4,619	[-10.797, 1.558]	0.138	
	Dichotomized PRUNE-S pain	5.504	[1.552, 9.455]	**0.008**	
Model 6	Smoking status (smoking is reference)	-11.038	[-17.075, -5.001]	** < 0.001**	0.367
	Snus use (current use is reference)	3.470	[-0.498, 7.437]	0.085	
	Preoperative symptom grade 1–5 (grade 1 is reference)	-1.087	[-2.381, 0.208]	0.097	
	Labor (non-manual labor is reference)	5.425	[1.335, 9,516]	**0.011**	
	Type of surgery (SD is reference)	-4.814	[-10.924, 1.296]	0.119	
	Dichotomized PRUNE-S pain	5.417	[1.549, 9.393]	**0.008**	

PRUNE-S = Patient-Rated Ulnar Nerve Evaluation Swedish version. SD = simple decompression. Statistically significant values (p < 0.05) are marked **in bold.**

## Discussion

In this prospective study of RTW after UNE surgery, predictive factors for sick leave length were identified. The median time before RTW was 6 weeks [IQR 4], which is in accordance with national guidelines on sick leave after UNE surgery. Being a non-smoker predicted a shorter sick leave period after surgery, while having manual labor and a higher degree of preoperative pain predicted a prolonged time before RTW following surgery.

The association between manual work and longer time before RTW would seem logical due to higher biomechanical demands in manual occupations, including work tasks such as holding tools and repetitive hand movements. While occupational factors may influence the onset of UNE, the type of work may not contribute to worse outcomes after surgery^11,18^. Following a traumatic upper limb nerve injury, manual laborers need a longer time before RTW compared to patients with non-manual labor^19^. Current Swedish national guidelines generally recommend a potential need for sick leave for up to 8 weeks (full or part-time) after UNE surgery, depending on the surgery and work tasks^8^. According to the prevailing clinical practice, however, patients undergoing simple decompression are likely to RTW at a median time of 6 weeks, irrespective of occupation^3^. The time before RTW showed a great variance in the present study, emphasizing the need for an individual assessment.

Smoking, but not snus use, was independently associated with longer sick leave in the present study. A similar association has been reported following work-related hand injuries, where patients who smoke return to work later compared to non-smokers^20^. Smoking can increase the risk of UNE development and surgical complications, such as persistent pain and wound healing complications, which in turn may affect RTW^5,6,21^. The proportion of smokers in this cohort was slightly higher than in the general Swedish population in 2024 (5%), while snus use was notably higher in the study cohort compared to the general Swedish population (16%)^22^. Smoking was more prevalent among women, while snus use was more common in men, which is in accordance with the frequencies in the general Swedish Population^22^. The clinical relevance of the observed sex difference regarding smoking should be interpreted with caution due to the low number of smokers (8/63) and the relatively weak statistical significance. Nicotine-containing snus reduces the peripheral skin blood circulation, which in turn can affect the healing of the wound^23^. Both smoking and snus use may therefore hinder recovery in patients undergoing surgery for UNE, as peripheral circulation is crucial for optimal healing. Smoking increases the risk of complications after surgery in general, and smoking cessation programs reduce the risk of complications in orthopedic surgeries^24,25^. In Sweden, smoking is overrepresented in people with lower education, while snus use is more evenly distributed across socioeconomic groups^22^. Conclusions of the effect of smoking in the present study should be interpreted with caution, since several confounding factors, such as socioeconomic status, might influence the results.

Pain is a prominent feature seen in patients with persistent or recurrent UNE^26^, and chronic neuropathic pain can severely impact overall health and sleep quality^27–29^, which may further hinder the ability to RTW. In contrast to the present study, Bruyns et al. did not find “pain in the hand” to be a predictor of RTW in patients with traumatic nerve injuries^19^. The high presence of various comorbidities, such as neck or shoulder pain, as well as other nerve compression syndromes among patients in this cohort, may contribute to the results, as these comorbidities could independently influence both preoperative and postoperative pain levels. This effect, however, was not studied enough in this work, but would be of great interest in future work.

We did not find any evidence suggesting that age, sex, or preoperative symptom grading influenced the time before RTW after surgery. This is consistent with research on patients with severe hand injuries and occupational hand injuries, where age and sex did not influence the RTW^20,30^. Older age has, however, been suggested to predict a poorer work-related outcome in patients having surgery for CTS^31^. The type of surgery was hypothesized to be associated with the length of sick leave. Only primary procedures, i.e., simple decompression or anterior subcutaneous transposition (the latter in cases with pre- or perioperative subluxation of the ulnar nerve), were included in the present study; revision surgeries were thus excluded. This may not fully mirror the real-world scenarios, as revision surgeries are common in clinical practice^6^. In a retrospective study including primary simple decompression, primary subcutaneous or submuscular transposition, as well as secondary surgeries (both transposition and simple decompression), the median sick leave following anterior transposition or revision surgery was 8 weeks, with patients undergoing transposition or revision surgery being four times more likely to experience prolonged RTW compared to those with SD surgery^13^. However, the present study did not find any statistically significant differences between the type of surgery and RTW. Similar results have been shown in a network meta-analysis examining different surgical techniques and RTW; however, none of the included studies included transposition surgeries^32^.

Following surgery, the PRUNE-S scores showed a decreasing trend for all the subscales as well as the total score, indicating improved functionality and lesser disability after surgery. When dichotomizing the preoperative PRUNE-S scores, no differences were seen regarding time before RTW except for the pain-subscale; this difference was, however, not statistically significant. This may be due to the relatively small sample size in the present study.

This study focuses on full-time sick leave and not part-time sick leave, which is a limitation. A few patients reported part-time sick leave in this study, with varying degrees over time (for example 50%, followed by 25%), making statistical analyses difficult to perform due to insufficient power. Another key limitation is the small sample size, which makes it difficult to capture additional relevant predictors across different subgroups. Moreover, the present study did not specify different occupational exposures, which limits the interpretation of the results to general occupational groups. The RTW process can be complex and may be influenced by several factors beyond those studied here, such as workplace-related factors and the patient’s self-efficacy for RTW (i.e., the patient’s belief in their ability to resume and manage work despite health challenges^33^). Due to insufficient data, results from preoperative electrophysiology testing (EnEG and EMG) are not included in this study which comprises another limitation. While the inclusion criteria were relatively broad to accommodate a heterogeneous patient population, some patients with more complex psychological conditions and those requiring revision surgeries were not included. This may reduce the applicability of the observed findings to a wider population, particularly those with more complex health challenges. In some cases, exclusions were also made due to complicated surgical procedures or incorrect initial diagnosis, indicating the challenge of diagnosing UNE correctly, as previously mentioned. These observations underline the importance of thorough evaluation and careful decision-making when considering surgery.

From a clinical perspective, awareness of the present findings could support physicians in managing patients with UNE. Conflicting results on occupational influence and UNE emphasize the need for further research into its role in UNE recovery. Screening tools like the PRUNE questionnaire could aid in identifying patients at risk for prolonged recovery and facilitate patient discussions about future occupational suitability. Early preoperative interventions, including tailored rehabilitation and patient education, could reduce these risks and optimize planning for RTW. While manual laborers in the present study generally returned to work within 8 weeks, more occupation-specific national guidelines could enhance their applicability to different individual needs. Implementing and studying preoperative smoking cessation programs, along with targeting snus use, could play a crucial role in reducing complications and shortening sick leave in future research.

## Conclusions

This study identified manual labor, smoking, and preoperative pain as factors predicting prolonged sick leave following UNE surgery. It is important to consider preventive strategies like patient education, workplace adaptations, and screening tools to prevent long sick leave duration. While sick leave patterns are important, they are just one challenge faced by UNE patients. Future research should explore other biopsychosocial aspects to improve understanding and refine recommendations to better meet individual patient needs.

## Supplementary Information

Supplementary Information.

## Data Availability

The data that support the findings of this study are not publicly available due to legal and ethical restrictions. According to Swedish legislation, including the General Data Protection Regulation (GDPR, Regulation (EU) 2016/679) and the Swedish Ethical Review Act (SFS 2003:460), the sharing of personal or sensitive data is strictly regulated. The data supporting the findings of this study are available from Region Östergötland, but restrictions apply to their availability, as they were used under license for the current study and are not publicly available. However, data are available from the corresponding author upon reasonable request and with permission from the National Ethics Review Board.
