# Supplementary material for: A prospective study of predictors of return to work after surgery for ulnar nerve entrapment
Source: Sci Rep. 2025 Sep 29;15:33334. doi: 10.1038/s41598-025-21589-z (PMC12479791; doi:10.1038/s41598-025-21589-z)
Supplement: Supplementary file 1 — Supplementary Information. [file 41598_2025_21589_MOESM1_ESM.docx]

# Appendix


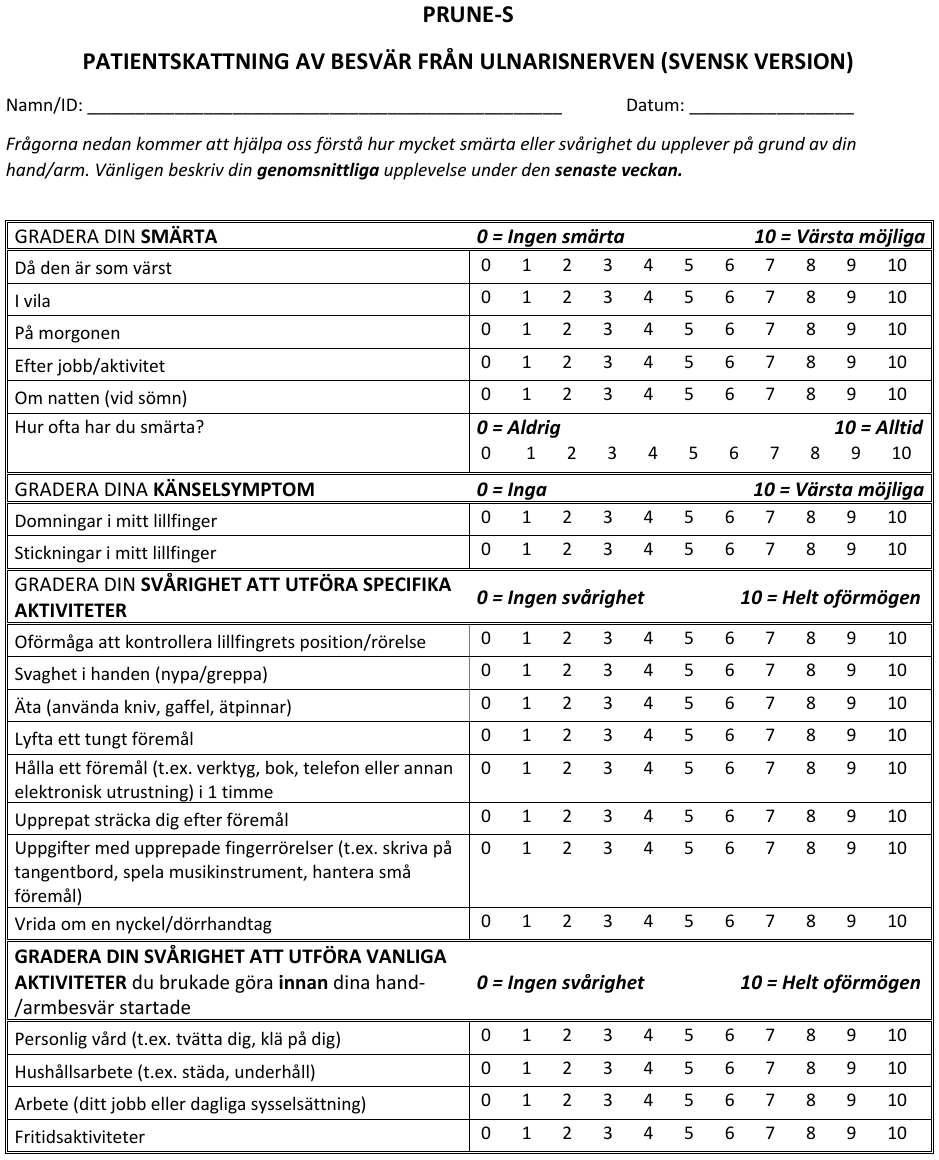
**Appendix 1. Swedish version of the Patient-Rated Ulnar Nerve Evaluation (PRUNE-S).**

The Patient-Rated Ulnar Nerve Evaluation (PRUNE) was developed by MacDermid et al (2013).

PRUNE-S was translated and validated by Papadopoulou et al. 2025 (Papadopoulou, A., Carlsson, I. K., Fornander, L., Dahlin, L. B. & Nyman, E. Translation and Validation of the Swedish Patient-Rated Ulnar Nerve Evaluation in Ulnar Nerve Entrapment. Plastic and Reconstructive Surgery – Global Open 13, e7044 (2025)).

Scoring: Pain subscale; sum of 6 questions on “pain” (/60); Sensorimotor subscale: sum of 2 questions on “sensory symptoms” (/20); Specific activities subscale: sum of 8 questions on “difficulty” (/80); Usual activities subscale: sum of 4 questions on “everyday activities (/40). Total score = is the sum of all subscales divided by two. Changes in score reflect changes in symptoms.
